# Alkalinity enhancement with sodium hydroxide in coastal ocean waters

**DOI:** 10.1038/s41598-025-31606-w

**Published:** 2025-12-12

**Authors:** Cathryn A. Wynn-Edwards, Wayne D.N. Dillon, John Akl, Craig Neill, Harris J. Anderson, Hui Sheng Lim, Mathieu Mongin, Elizabeth H. Shadwick

**Affiliations:** 1 CSIRO, Environment, Hobart, 7000 Australia; 2 CSIRO, NCMI, Hobart, 7000 Australia

**Keywords:** Ocean Alkalinity Enhancement, mCDR, Sodium Hydroxide, Field Trial, Coastal, Australia, Climate sciences, Environmental sciences, Ocean sciences

## Abstract

Carbon Dioxide Removal (CDR) is increasingly recognised as essential for achieving net zero emissions to limit the impacts of climate change. Ocean Alkalinity Enhancement (OAE) presents a potentially scalable marine CDR (mCDR) technique. Here we report on the first OAE field trial in Australia, conducted at a coastal site in Tasmania using continuous addition of aqueous sodium hydroxide (NaOH). The resulting plume of modified seawater was effectively tracked, and changes in surface carbonate chemistry were quantified using a containerised laboratory. At the point of NaOH release, partial pressure of CO$_2$ (*p*CO$_2$) decreased by up to 370 $\mu$atm with alkalinity increasing by approximately 545 $\mu$mol kg$^{-1}$. Maximum downstream decreases in *p*CO$_2$ ranged from 22 to 77 $\mu$atm, corresponding to signal strengths of < 1 - 5$\%$. This small-scale field trial confirmed that the dispersion of a plume of modified seawater occurs rapidly and within meters of the site of addition, and that with appropriate tools, these changes can be measured directly in a coastal ocean location. These results suggest that the deployment of shore-based OAE, in combination with local coastal infrastructure and regional models, have potential as an mCDR approach.

## Introduction

The impacts of climate change are increasingly obvious and severe. Limiting global warming to 1.5 $^{\circ }$C, was the ambition of the 2015 Paris Agreement^1^, however achieving this by reducing greenhouse gas emissions alone is no longer possible; Carbon Dioxide Removal (CDR) is required to limit warming to 2 $^{\circ }$C and to counterbalance hard-to-abate emissions (from agriculture, aviation and other industries)^2^. Land-based CDR technologies range from afforestation and reforestation, to direct air capture, and enhanced weathering^3,4^. Ocean based, or marine CDR (mCDR) methods include artificial upwelling, ocean fertilization, biomass sinking and ocean alkalinity enhancement^2,5–7^. The ocean has already absorbed an estimated 25% of anthropogenic CO$_2$ emissions^8^, and is the largest reservoir of carbon interacting with the atmosphere, with the majority of the roughly 38,000 Gt carbon in the form of total dissolved inorganic carbon (TCO$_2$). When CO$_2$ reacts with seawater it is quickly converted to bicarbonate (HCO$_3^{-}$) and carbonate (CO$_{3}^{-2}$) ions, which are stable and have long (>10,000 yrs) residence times in the ocean; the extent of this conversion is governed by the buffering capacity of the ocean, or its alkalinity (Alk).

**Fig. 1:**
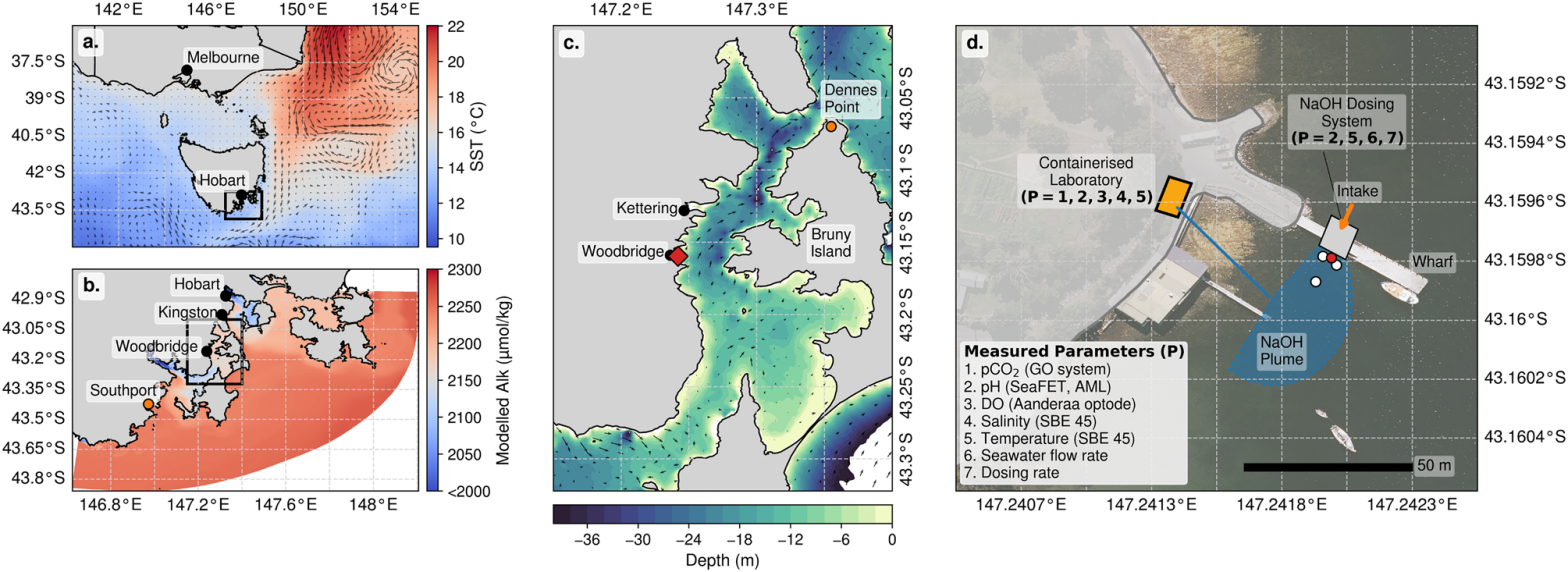
Study site and experimental design. **a**. sea surface temperature (SST) for the broader Tasmanian region^68^. **b**. surface Alk from a high resolution biogeochemical model^29^ in the coastal ocean south-east of Tasmania (region in the black box in panel **a**). **c**. bathymetry of the study region^69^ near Woodbridge Tasmania (red diamond). **d**. schematic representation of the field trial location overlaid on aerial photograph^70^, associated sampling locations and parameters measured during the experiments. This figure was created using Python v3.9.23 (https://www.python.org/) and matplotlib v3.9.4 (https://matplotlib.org/).

Alkalinity is naturally added to the oceans by the weathering of rocks; enhancing this process to increase the capacity of the ocean to absorb CO$_2$, and store it as TCO$_2$, has garnered interest as an mCDR method referred to as Ocean Alkalinity Enhancement (OAE)^9–12^. Increasingly, OAE research is moving from laboratory experiments^13–16^ to field trials^12,17–20^, with a focus on feasibility, efficiency and potential ecological impacts. While more research is required, early work indicates that the ecological impacts of OAE at magnitudes that are realistic for large scale deployment, especially via non-mineral Alk sources, are modest, particularly in light of the climatic benefits and the consequences of unabated climate change^15,18,19,21,22^. By 2050, Australia will need to abate 133 Mt CO$_2$ yr$^{-1}$, or roughly 24$\%$ of its 2005 emissions^23^. Given Australia’s extensive coast line and exclusive economic zone (EEZ, globally, the third largest by area), mCDR technologies from shore-based facilities hold great potential, with the southernmost state of Tasmania associated with the greatest potential for efficiency^24^. Here we present the first deliberate OAE field experiment focused on mCDR in Australia (see Albright *et al.*^25^ for the purpose of reversing ocean acidification) using aqueous sodium hydroxide (NaOH) in a coastal ocean location (Fig. 1). Alk can be added in aqueous form (e.g. NaOH or magnesium hydroxide (Mg(OH)$_2$) by chemical dissolution or electrochemically produced Alk) or solid form, using minerals such as olivine or brucite^13,15–18,21,26–28^. Here we demonstrate the functionality of a novel dosing system to deliver aqueous NaOH and a largely automated system used to track the plume of modified seawater and quantify the short-term changes in carbonate chemistry. We chose aqueous NaOH as the source of Alk since it has the least potential to disrupt biogeochemical cycles^15,18,21^. An aqueous solution also allows for accurate dosing of added Alk at a known concentration. This small-scale trial (modified seawater plume on the order of 25 m$^2$) was not intended to achieve measurable mCDR, but rather to serve as a proof-of-concept for the safe (i.e., with an emphasis on controlled, localised pH perturbations, demonstrating rapid dilution and return to defined baseline conditions), and efficient deployment of a shore-based OAE technology and the required tools to monitor carbonate system perturbations in the coastal ocean. The results of this field trial are linked to the study of Anderson *et al.*^29^ that used an ocean circulation model to quantify how added Alk might disperse in this region and its effectiveness in sequestering carbon dioxide, especially when the Alk is produced from renewable energy sources and existing coastal infrastructure is utilized. The Australian state of Tasmania is therefore well positioned since almost 100$\%$ of its electricity production is based on renewable energy^30^.

## Results

**Fig. 2:**
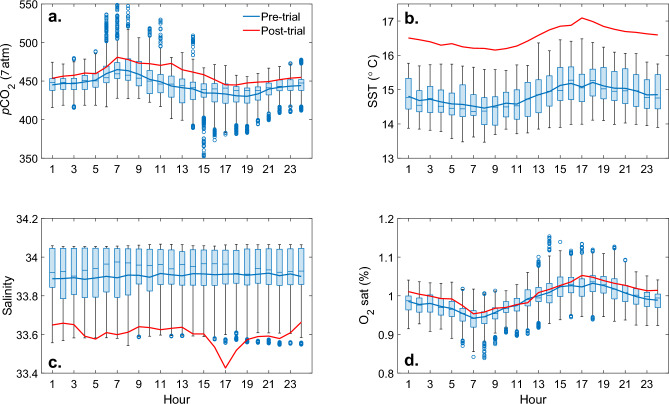
Pre- and post-trial hydrographic and biogeochemical observations **a**. *p*CO$_2$. **b**. sea surface temperature (SST). **c**. salinity. **d**. dissolved oxygen saturation (O$_2$sat). The blue (pre-trial) and red (post-trial) lines in all panels are the hourly means of the observations. While the local hydrography changed (see also Fig. S1), consistent with the seasonal transition at this time of year, post-trial *p*CO$_2$ remained within the variability observed prior to the experiment.

Baseline observations were collected prior to (3 weeks for CO$_2$ partial pressure, *p*CO$_2$, and 10 days for pH), and one week following, the field trial (Figs. 2 and S1 and Table S1). Mean (standard deviation) sea surface temperature (SST) and salinity were 15 $^{\circ }$C (± 1.0), and 34 (± 0.2), respectively, with *p*CO$_2$ supersaturation (449 $\mu$atm ± 20) reflecting the estuarine nature of the D’Entrecasteaux Channel (Fig. 1). The mean concentrations of TCO$_2$ and Alk were 1976 $\mu$mol kg$^{-1}$ (± 41) and 2152 $\mu$mol kg$^{-1}$ (± 46), respectively, and mean dissolved oxygen saturation (O$_2$sat) was 99$\%$ (± 4). Mean diurnal variation in *p*CO$_2$ was $\sim$ 77 $\mu$atm (± 25), with a maximum diurnal signal of $\sim$ 121 $\mu$atm (Fig. 2). The anti-correlation between observations of O$_2$sat and *p*CO$_2$ (Fig. S2) confirms that biology dominates short term variations in the CO$_2$ system in the shallow bay in austral spring.

During the field trial, aqueous NaOH was released to the ocean using an automated dosing system (see Methods). The NaOH was added at a rate of 15 L hr$^{-1}$ to a stream of seawater collected from the northern side of the jetty (see Fig. 1d.) with a submersible pump, and re-released on the southern side of the jetty at a rate of $\sim$ 13,250 L hr$^{-1}$. Four pseudo-replicate experiments were conducted over two consecutive days (25 and 26 November, 2024). The total volume of treated seawater was similar on both days ($\sim$ 37,000 and 50,000 L) and with an average NaOH enrichment of 1091 $\mu$mol kg$^{-1}$.

**Fig. 3:**
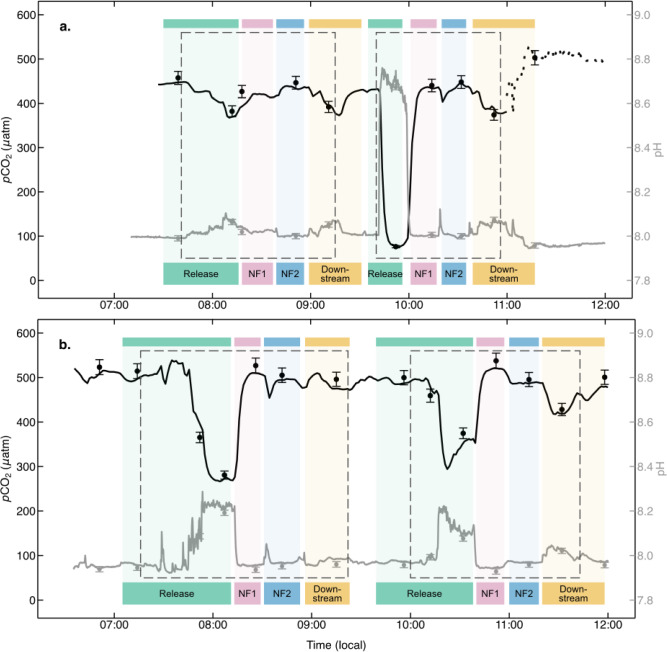
The addition of 1M NaOH solution to the coastal ocean at Woodbridge. Time series observations of *p*CO$_2$ (± 2 $\mu$atm) and pH (± 0.24) during experimental trials on Day 1 (**a**) and Day 2 (**b**) with the locations of observations (release, near field (NF1 and NF2), downstream) given by the shading and labelled at the bottom of the panels. The periods of NaOH release are enclosed by the dashed boxes. Also shown as points are the *p*CO$_2$ and pH values calculated from the discrete TCO$_2$ and Alk samples, with error bars representing their respective uncertainties of ± 14 $\mu$atm and ± 0.012. On Day 1, the *p*CO$_2$ observations were not recorded after 11:00, and have been estimated (dashed black line) on the basis of the corresponding pH observations, and the Alk sample (assumed constant in time) collected just prior.

**Fig. 4:**
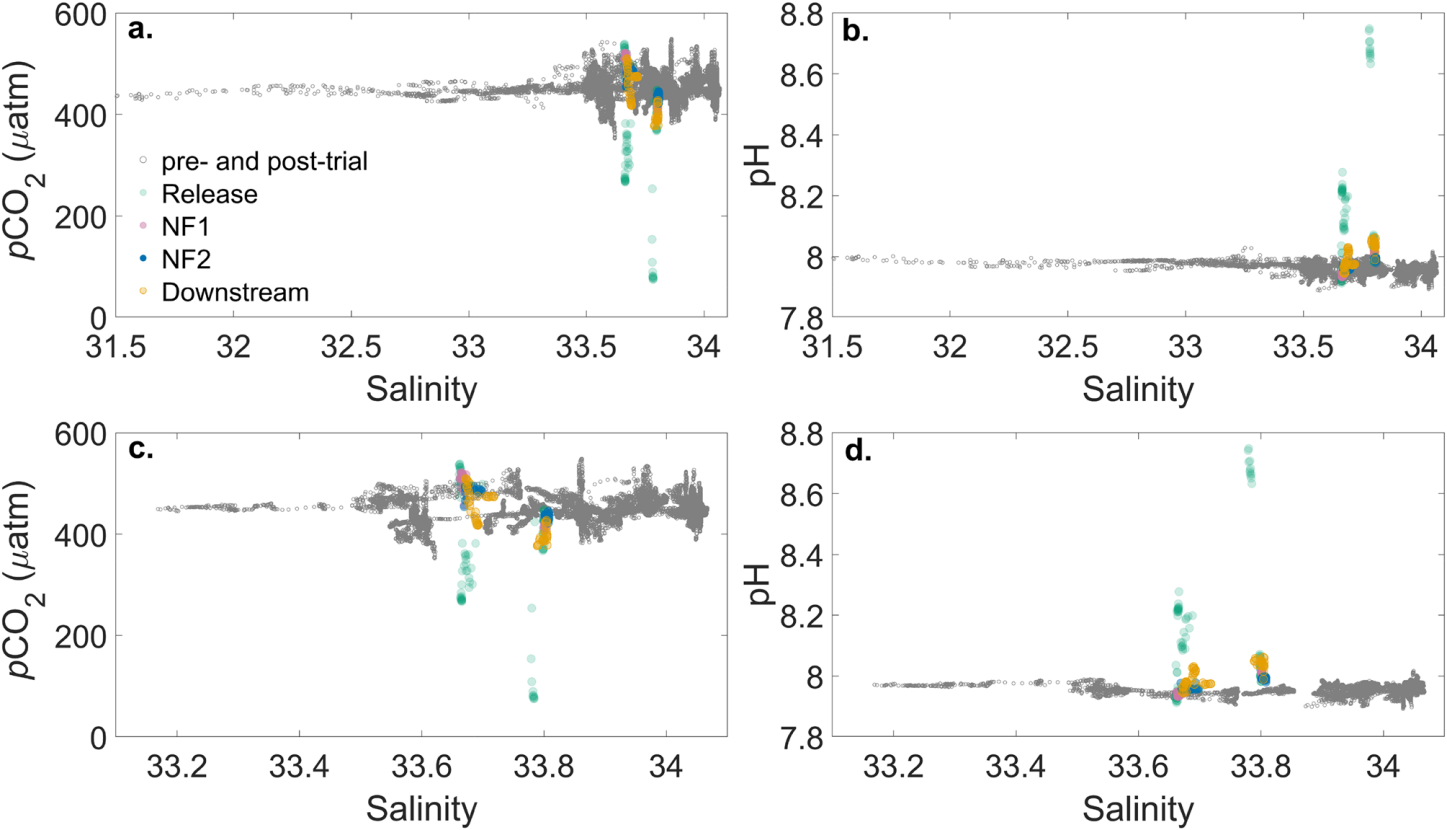
Variation of the CO$_2$ system with salinity at the field trial site. **a**. *p*CO$_2$ (± 2 $\mu$atm) versus salinity all observations collected prior to, during, and following the experimental trial; **b**. pH (± 0.012) versus salinity as in panel **a**; **c**. *p*CO$_2$ versus salinity (± 0.01) for observations collected only during the same hours as the NaOH release trials (08:00 - 12:00 local time). **d**. pH versus salinity (± 0.01) as in panel **c.** In all panels, the gray data are observations from pre- and post-trial, while the coloured symbols indicate observations collected during the NaOH release at the release, near field (NF), and downstream sampling locations (as in Fig. 3).

**Fig. 5:**
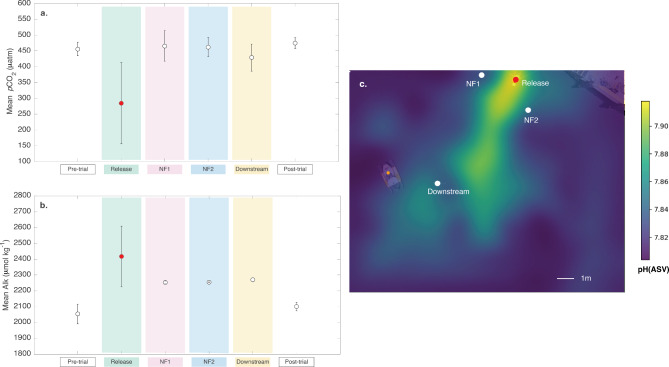
Observations within and outside the NaOH plume. **a**. mean *p*CO$_2$ (with standard deviation) for the pre- and post-trial observations, as well as observations collected in the immediate vicinity of the release, and at the near-field (NF), and downstream sampling locations. **b**. mean Alk (with standard deviation) as in panel **a**. **c**. the variation in surface ocean pH(ASV) mapped with a piloted vehicle during Day 2 of the experiment. We note there is an offset between the pH(ASV) and the pH observations shown in Figs. 3 and 4. The pH(ASV) is used here as a proxy to illustrate spatial changes, and not as an independent measurement of pH.

During the first release on the first experimental day, the NaOH addition resulted in a large decrease in *p*CO$_2$ at the release position (*p* = 0.17). The corresponding increase in pH and Alk (Fig. 3, 4 and 5) relative to the multi-week pre-experimental mean were not significant. Over the two consecutive days, there was a consistent reduction in *p*CO$_2$ at the release position (Fig. 1d. and 3), relative to both the immediate (minutes) pre-dosing observations, and the downstream, and near field (NF1 and NF2) locations, with a maximum reduction ranging from 78 to 370 $\mu$atm during dosing. There was a concomitant increase in pH from the one week pre-trial baseline mean value of 7.98 ± 0.01 to a maximum of 8.76 (i.e., a 0.77 increase relative to the immediate pre-dosing pH), with the largest pH change observed in the immediate vicinity of the release (Fig. 3 and 5). There was no measurable change in TCO$_2$ during the experiment, relative to the immediate pre-trial discrete samples, and no observed difference in TCO$_2$ between the vicinity of the release and the downstream location (Table 1), confirming the expectation of conservation of mass for TCO$_2$ (i.e. a shift in CO$_2$ speciation due to the increased Alk leads to an increase in $\mathrm HCO_3{^-}$ and CO$_3^{2-}$ but the total TCO$_2$ remains unchanged^31,32^). However, as expected, there was an enhancement in Alk during the experiment, with a maximum increase of 544 $\mu$mol kg$^{-1}$ at the release, and 49 $\mu$mol kg$^{-1}$ downstream during the first release on the first experimental day, relative to immediate pre-trial discrete sample (Table 1).

While the changes in the carbonate system described above were detectable in the immediate vicinity of the release, and to a lesser extent at the other sampling locations, the NaOH signal was diluted over small spatial scales (meters, see Fig. 5). The signal strength (Table 1) illustrates how much of the pure dosed seawater was detected at each measurement location (based on added NaOH and measured Alk), and indicates that dilution occurred rapidly over $\sim$7 meters (i.e., the distance between the release and downstream sampling locations) to between 0.6 and 5 $\%$ of the full signal (see Table 1). Furthermore, mean observations of *p*CO$_2$, Alk, and pH at the NF1, NF2 and downstream locations were not significantly different from mean pre- and post-trial observations, and therefore within the range of the natural variability observed over several weeks at the field trial location (Figs. 4 and 5). We note that the TCO$_2$ samples collected during the experiment were however elevated relative to the mean pre- and post-trial values, which were computed from paired *p*CO$_2$ and pH sensor observations (see Methods), and therefore have larger associated uncertainty than the discrete samples collected during the experiment.

In addition, the change in *p*CO$_2$ at all locations except for the immediate vicinity of the release, while attributable to the Alk enhancement, was much smaller than the natural variability in the system (Fig. 4), which from a regulatory and social perspective^33^, may make coastal locations attractive as it is likely that the biological community has adapted to cope with high frequency variability in ocean chemistry^34,35^. Furthermore, the recovery of the system between NaOH additions, as well as at the conclusion of the trials, was rapid, with a return to baseline conditions within an hour after dosing concluded (Fig. 2 and 3).

## Discussion

We conducted a field trial of OAE to test an operational in-situ system for chemical release and carbon chemistry measurements. The location, timing, and scale of the experiment were selected based on infrastructure availability, an analysis of local water circulation and weather patterns^29^, regulatory and ecological considerations, and social constraints^33^. Our results and analysis show that this strategy was successful and should inform future experiments that might be conducted at larger scales.

We used autonomous sensors to measure both *p*CO$_2$ and pH continuously (before, after and during the field trials), and collected discrete TCO$_2$ and Alk samples for sensor validation (Fig. 3, see Methods). We found good internal consistency in our CO$_2$ system observations: the mean differences between in situ and predicted values were -12.9 ± 15.9 $\mu$atm for *p*CO$_2$ and -0.02±0.06 for pH, with zero falling within one standard deviation for both, indicating no statistically significant bias between measured and estimated values. This reinforces the recent community consensus that *p*CO$_2$ and pH are suitable CO$_2$ system parameters for in situ OAE monitoring^5,36^, assuming that the sensors deployed in the field return data of sufficient quality, and are validated with bench top measurement of TCO$_2$ and Alk^31,37^.

The experimental design presented here included a number of advantages. The first is the use of the bespoke system for NaOH addition to a stream of local seawater (see Methods). This dosing system, which was tested in a closed loop prior to use in our trials, allowed the experiment to be undertaken with relatively small volumes of concentrated NaOH. By using a submersible pump to release large volumes of dosed seawater, no pre-mixing of large volumes or the transport and movement of holding tanks was required (as was done, for example by Albright *et al.*^25^). Additionally, by choosing to conduct trials in the lead up to and/or during the outgoing tide, and only in low wind conditions, seawater could be pumped from one side of the jetty, then subsequently dosed with NaOH and released on the other side, without a possible re-entrainment of previously perturbed water, or inadvertent re-dosing of the same water.

The dosing system also included a pH sensor at the outlet (see Fig. S3), and a safety shut down at a pH > 9.0, which could be used to ensure operation within the predetermined ecologically safe limit (toxicity results not shown) and to eliminate the chance of precipitation^16,22,26^. The second advantage, was the use of an on-site laboratory facility that allowed observations from discrete locations (release, NF1, NF2, and downstream) to be acquired continuously for both *p*CO$_2$ and pH, yielding high quality measurements, both within, and outside, the plume of modified seawater (with the intake to the container relocated in sequence to each of the fixed locations). The same set of instrumentation was used to collect baseline observations prior to, and upon completion of, the NaOH release experiments (see Methods). Such a system could be used for larger scale OAE deployments from fixed coastal infrastructure (discussed below), ideally, with the addition of a moored sensor in a fixed location downstream of the plume of modified seawater such that observations could be collected simultaneously, which was not achieved here. Additionally, because we were unable to co-locate our pH sensor with the intake to the *p*CO$_2$ sensor, the observations were not collected at the same temperature, and therefore required correction of the pH data to the in-situ water temperature. This step, while straight forward with CO2sys^38^, does result in a pH time series with significantly larger uncertainty than the corresponding *p*CO$_2$. Moreover, in a larger scale deployment, it is unlikely that the *p*CO$_2$ measurements at either the release or downstream location would be made with a direct air–water equilibration system, as a lower cost membrane-based sensor would provide sufficient quality observations for mCDR applications^39^.

The rapid signal dilution, in both space and time, is consistent with the analysis of model simulations in the region^29^. In addition, the modelling study of Anderson *et al.*^29^ evaluated simulated OAE at the study site using a rate of Alk addition of 2.45 mol s$^{-1}$ in 1-hour simulations, which is roughly 600 times larger than the perturbation achieved with the NaOH release presented here. The Anderson *et al.*^29^ simulations were designed, in part, to inform this field trial, and indicated that simulated short-term Alk release resulted in plumes on the order of 1 km$^2$ (with model grid cell size of 200 - 300 m), which dispersed over time scales of hours, with simulated pH perturbations below the detection limit (pH < 0.003) at these scales. The field trial, which used significantly lower rates of Alk addition, has shown that the pH at the release is much higher than in the model, due to the small spatial scale of 'real world’ experiment. The field trial has also highlighted some limitations of the model in predicting detectability, as we are reporting observations of features here that cannot be represented in a model with 200 m resolution. Finally, the field trial indicates that the model pH threshold of 0.003 as a detectability limit is likely unsuitable.

The mean air-sea flux of CO$_2$ over the three weeks prior to the experimental trials was 1.05 mmol m$^{-2}$ d$^{-1}$, with positive values indicating that the ocean was outgassing CO$_2$ to the atmosphere, which is common in estuarine and near shore environments^40^. While the small-scale, brief, proof-of-concept trial presented here did not result in detectable additional uptake of CO$_2$ from the atmosphere or a reduction in the amount of CO$_2$ outgassing (which would nevertheless represent mCDR), it is possible to estimate the impact such a perturbation would have if it were conducted at a larger scale and/or over a longer period. In the theoretical case of a continuous dosing of NaOH, and assuming the maximum *p*CO$_2$ decrease (370 $\mu$atm) we observed during our trials was induced and remained constant, the air-sea flux of CO$_2$ over a three-week period would be on the order of -11.74 mmol m$^{-2}$ d$^{-1}$, with this estuarine region converted to a net sink for atmospheric CO$_2$ in austral spring. Our goal here is not, however, to make a quantitative estimate of the local mCDR potential on the basis of our observations, but rather to emphasize that such potential exists, and that available tools can be used to undertake controlled deployment of OAE.

## Conclusion and outlook

To evaluate the feasibility of OAE at scale, field experiments, of increasing size and duration, are required to establish ranges of natural variability in coastal locations, to quantify limits of detection, and to demonstrate the ability to track and monitor the deliberate perturbation of seawater chemistry. Our successful field trial has demonstrated that such monitoring can be undertaken with existing sensors, and potentially without the need for a tracer dye^41^. Integrating the results of this small scale field trial with high-resolution biogeochemical model results for the same region remains challenging, and additional effort to better match the spatial and temporal scales of the observations and simulations is needed.

While studies conducted so far have indicated that Alk addition of realistic magnitude for deployments at scale cause modest ecological responses, acknowledging winners and losers^15,18,19,21,22,42,43^, future work should include ecological monitoring with emphasis on the distinction between impacts at point-source concentrations and those downstream or in the far field. Longer term experiments with comprehensive monitoring prior to, and after, Alk perturbations are also needed. Furthermore, local conditions such as the load of suspended sediments and the potential for interactions with sedimentary Alk^12,16^ should also be considered. Finally, an appreciation for the diversity of stakeholder perspectives and objectives is vital, and consultation with local communities should be considered a requirement of all future work^33,44,45^.

One of the limitations of using OAE at scale is the energy required for, and therefore cost associated with, the production of alkaline solutions or materials^46,47^ suitable for release in the ocean^5,48,49^. While more expensive^48^, electrochemical methods, which use the splitting of seawater into acidic (e.g., hydrochloric acid, HCl), and basic (e.g., NaOH) solutions^26,49–51^, may potentially be associated with the smallest environmental impacts of OAE^11,18^. The Australian state of Tasmania is powered almost entirely by renewable energy, through a combination of hydro-electric and wind-powered electricity generation^30^, making it an attractive location for the deployment of electrochemical mCDR at scale. A global assessment of the potential for converting renewable energy for CDR, resulted in a range of 0.15-0.22 Gt CO$_2$ removed per EJ (Exa Joules, 10$^{18}$)^49^. While this is a global estimate, which was not specific to the configuration of OAE and does not take into account the varying efficiency of OAE deployment due to regional geography or the limitations of the permitting environment, it can be used to gauge the CDR potential for Tasmania via OAE from renewable energy production. Combining the range of energy requirement per Gt of CO$_2$ removed, (i.e., 0.15-0.22 Gt CO$_2$ removed per EJ^49^) with an assumed use of 1$\%$ of Tasmania’s annual renewable energy production (0.0033 EJ), results in a potential mCDR range of 0.50 to 0.73 Mt CO$_2$ yr$^{-1}$. This corresponds to 6 to 9$\%$ of Tasmania’s annual emissions (8.17 Mt CO$_2$ from energy production, industrial processes, agriculture and waste^30^) removed by OAE. There is an emerging consensus that the deployment of OAE at scales required for meaningful contributions to CDR will require the use of existing coastal infrastructure, used, for example, by wastewater treatment^17^ or desalination plants^52,53^. Wastewater treatment plants provide the infrastructure to release large volumes of water for dilution of added Alk, and hence minimizing the chance of secondary precipitation, while the added Alk reduces the amount of CO$_2$ outgassing from the released waste water stream^17,26^. Based on the annual report by the state water and sewage management corporation, TasWater, each sewage wastewater treatment plant processes roughly 450 million L of water per year^54^. We discharged an average of 13,000 L hr$^{-1}$, or 116 million L yr$^{-1}$, of modified seawater during our experimental trials, which indicates that any one of the 110 treatment plants currently operating in the state could support a deployment of OAE at the scales required for quantifiable mCDR in Tasmania, a promising next step in assessing the feasibility of this climate solution.

## Methods

### Experimental setting

The field site was a small bay ($\sim$ 4.5 km$^{2}$) at Woodbridge, Tasmania, 43$^\circ$S, 147$^\circ$E, located within the D’Entrecasteaux Channel (Fig. 1). Water depth in the Channel between the two headlands of the bay is $\sim$ 8 m. Site location criteria included logistics (proximity to the laboratory in Hobart, a secure site with electricity), the sheltered character of the site and the existence of a hydrodynamic and biogeochemical model for the area^29^. The aim was to conduct four pseudo-replicate Alk releases on two consecutive days. A short break between releases on each day would ensure a return to baseline of measurable parameters and therefore assure that the pseudo-replicates would be independent. To achieve comparable and optimal hydrodynamic conditions, additional criteria for the timing of the experiment were related to known factors that increase the efficiency of the added Alk, such as season (optimum in austral summer vs winter), tidal cycle and circulation patterns and atmospheric conditions (such as wind-speed)^16,29,55^. Therefore, the Alk releases were restricted to early hours of the day to reduce the influence of wind-driven advection on plume dispersion. Specifically, experimental days were chosen such that the outgoing tide was at least 3 hours later than the planned (early morning) start time. This meant that the submersible pump (see below) could be used alongside the jetty, in sufficient water depth, with the dominant flow to the south such that seawater collected on the northern side of the jetty carried no (or little) risk of re-entrainment of modified seawater during release of dosed seawater on the southern side of the jetty. As such, the experiment was conducted on two consecutive days (Nov 25 and 26, 2024) on which high tide peaked during the early daylight hours (trials began between 07:00 am and 08:00 am local time). The week before the experiment was conducted, a test run of the NaOH dosing system (described below) was undertaken using freshwater (i.e., with no added NaOH). Changes in salinity on the order of 0.05 kg m$^{-3}$ were observed between 2 and 3 m from the freshwater release. Additionally, idealised one dimensional advection-diffusion modelling indicated that an addition at a $\mathrm pH = 9$ would result in a pH increase of 0.25 (from a background of $\mathrm pH = 8$) in approximately 7 minutes at a distance of 5 m (see Fig. S4). This informed the choice of four sampling locations: 1. release (immediate vicinity of the dosing system outlet); 2. near-field 1, NF1 (roughly 2 m offshore, or east, of the outlet); 3. near-field 2, NF2 (roughly 2 m inshore, or west, of the outlet); and 4. beyond near-field, hereafter called downstream (6.5 m, or roughly twice the footprint of the freshwater signal, south of the outlet). During the experiments the intake cage (see below) with the sensors was moved from station to station with a small boat to measure the signal of the dosed seawater plume at an approximate water depth of $\sim$ 0.5 m.

### CO$_2$ system observations

Baseline observations of seawater temperature, salinity, *p*CO$_2$, pH and dissolved oxygen were acquired using a containerised laboratory for three weeks leading up to, and one week following, the experiments. Seawater intake to the container was via a 50 m insulated hose. A stainless steel cage protected the intake and housed a temperature sensor (SBE39plus). Inside the container, an underway system was used to measure seawater and atmospheric xCO$_2$^56^ with minor modifications outlined in^57^. Measured seawater xCO$_2$ was converted to *p*CO$_2$ via the intake temperature measurements, to account for the warming of the seawater along the 50 m intake hose and the heat generated by the pump inside the container. The temperature correction was done via the empirical relationship of Takahashi *et al.*^58^ and the measurements from the containerised laboratory were aligned in time with seawater temperature measurements at the intake cage based on the measured flow rate through the intake line. The uncertainty in seawater *p*CO$_2$ measurements with this system is ± 2$\mu$atm^39,57^.

Atmospheric xCO$_2$ was measured approximately 3 meters above the high tide sea level, and no correction to sea level was applied. Additionally, atmospheric xCO$_2$ values exceeding mean monthly values from measured at the Kennaook/Cape Grim Baseline Air Pollution Station^59^ (56$\%$) were excluded, as the air intake was located near commercial and residential infrastructure, where potential contamination could not be verified during measurements. A linear interpolation was used to fill gaps from excluded or missing data. The uncertainty of the atmospheric *p*CO$_2$ is ± 0.2$\mu$atm^39,57^. Given the slow equilibration time of CO$_2$ (weeks to months), the linear interpolation and consequent potential smoothing over short-lived atmospheric *p*CO$_2$ perturbation introduces a small error compared to the known uncertainty that stems for example from wind-speed parametrizations^60^.

Air temperature and wind-speed data at minute resolution was sourced from the Australian Bureau of Meteorology weather station at Dennes Point (on the northern tip of Bruny Island, Fig. 1c.). Tide data was sourced from the Australian Bureau of Meteorology tide gauge at Southport (Fig. 1b.).

The pH was measured using an SBE SeapHox sensor, calibrated using discrete seawater samples collected alongside the sensor (see below). The residuals (difference between the calculated pH and the SeaFET pH) from three replicate calibrations were: 0.0895, 0.085, and 0.085, yielding a mean offset (bias) of 0.0865. The standard deviation of the residuals, representing the random error of the SeaFET, was 0.0026. The SeaFET readings were corrected for the measured bias, and the resulting uncertainty represents the accuracy of the calibrated sensor.

During the experiments, the intake to the container was moved between the four sampling locations with a small boat; the intake was fixed to a moored float during measurements, and the boat moved well outside (50 m) of the experimental area. Based on the intake pump speed and the length of the hose, the seawater signal took $\sim$ 3 min to be registered by the sensors inside the container. All data were gridded to 80 s intervals and data gaps (e.g. due to calibration of *p*CO$_2$ sensor, 12$\%$ of pre-experiment data and 2$\%$ of post-experiment data) linearly interpolated. Data collected during dosing were excluded before computing a 24-hr running mean.

Discrete water samples were taken at the start of each experimental day and before each trial (with the exception of trial two on day one), at every sampling location and at the end of the second trial on each day (see Fig. 3). Samples were analysed for concentrations of TCO$_2$ and Alk, following standard procedures^37^, and have associated uncertainty of ± 2 $\mu$mol kg$^{-1}$.

### Calculations and statistical analysis

Using discrete samples of TCO$_{2}$ and Alk collected during the experiment, pH (total scale) and *p*CO$_{2}$ were calculated for comparison with sensor data. Calculations were done with the CO2sys program^38^ using the carbonic acid dissociation constants of Lueker *et al.*^61^, the KF values of Perez and Fraga^62^, the KS values of Dickson^63^, and the Boron to salinity ratio of Uppström^64^. Phosphate and silicate concentrations were excluded from the calculations. The uncertainties of calculated carbonate parameters were estimated using the uncertainty propagation algorithm of Orr *et al.*^65^ assuming measurement uncertainties of ± 2 $\mu$mol kg$^{-1}$ in both TCO$_{2}$ and Alk, ± 0.01 in salinity, and ± 0.01 $^{\circ }$C in temperature.

Because the *p*CO$_2$ and pH sensor were not immediately co-located and thus not measuring at the same temperature, a temperature correction after Takahashi *et al.*^58^ was used to bring *p*CO$_2$ to a common temperature of the pH measurement. The pH values were then corrected to the in-situ temperature with this temperature adjusted *p*CO$_2$. The propagated uncertainty yields a mean uncertainty of 0.24 in pH, which is much larger than the sensor accuracy and primarily reflects the inherent limitations of the *p*CO$_2$ – pH pair used for carbonate system characterisation. The (corrected) pH was used with *p*CO$_2$ to calculate pre- and post-trial background Alk and TCO$_2$, with a mean uncertainty estimate of ± 69 $\mu$mol kg$^{-1}$ and ± 78 $\mu$mol kg$^{-1}$, respectively.

The air-sea flux of CO$_2$ was computed using the equation below,

1 $$\begin{aligned} FCO_2 = k \alpha \Delta \textit{p}CO_2 \end{aligned}$$

where k is the gas transfer velocity, with a scaling factor for CCMP2 winds, according to^66^ for better comparison with literature values, however we did use local minute resolution wind-speed data. $\alpha$ is the coefficient of CO$_2$ solubility according to^67^ and $\Delta$*p*CO$_2$ is the gradient between seawater *p*CO$_2$ and atmospheric *p*CO$_2$ such that negative FCO$_2$ indicates CO$_2$ uptake by the ocean. Using Kennaook / Cape Grim monthly mean *p*CO$_2$ data yielded indistinguishable results.

To evaluate whether mean seawater *p*CO$_2$, TCO$_2$, TA and pH differed among positions during dosing and pre- and post-experiment multi-week baseline means, we fitted a linear mixed-effects model of the form

2 $$\begin{aligned} Mean Response_{ij} = \beta _0 + \beta _{Groupi} + b_j + \epsilon _{ij} \end{aligned}$$

where *Group* (Pre, Post, and each Position $\times$ Day combination) was treated as a fixed effect and *Block* (the combination of sampling day and replicate sequence) as a random intercept to account for repeated sampling structure. The model was fitted using restricted maximum likelihood estimation and the pre-experiment means served as the baseline (reference level). The random term b$_j$ $\sim$ N(0,$\sigma ^{2}_{b}$) captures variability among blocks, while the residual term $\epsilon _{ij}$ $\sim$ N(0,$\sigma$ $^{2}$) represents within-block variation. An overall F-test from the model ANOVA table assessed whether group-level differences were significant. To identify specific contrasts, we used linear hypothesis testing on the fitted model coefficients to compute *p*-values for pairwise comparisons with both the pre- and post-experiment means. These post-hoc contrasts were constructed manually from the model coefficient vector to evaluate targeted differences among groups.

### NaOH dosing system

An inline dosing system (Fig. S3) was designed to facilitate automated additions of an aqueous NaOH solution to a continuous flow of locally sourced seawater. Constant feedback from a pH sensor at the outflow enabled dynamic control of the dosing rate by the operating software, ensuring that the modified seawater was kept under pH = 9, to eliminate the risk of precipitation and negative ecological impacts^16,22,26^. In practice during the trial, a suitable pH was achieved with a dosing rate of $\sim$ 15 L hr$^{-1}$ 1 M NaOH solution. The NaOH dosing rate was constant for all runs except the first, during which the rate was adjusted to identify the optimal flow. Seawater flow rates varied slightly between runs, and the low standard deviations indicate consistent and comparable conditions across experiments (Table S2). A description of the system’s plumbing hardware, computer software, and communications follows.

**Plumbing hardware** A submersible pump continuously pumped seawater through the system, which consisted of an inline flow meter and a static mixer tube connected by 2” hosing (Table S3). The controlled addition NaOH was facilitated by a dosing pump with an operational range of 0 – 200 L hr$^{-1}$. The output of the dosing pump (3/8” tubing) was split into four separate streams (1/4” tubing) using a series of successive Push-To-Connect Y fittings (i.e., the two streams from an initial Y split were each split again). This dispersion of the NaOH solution was designed to minimise localised spikes in seawater pH, reducing risk of precipitation. Effective mixing was indicated by the stable outlet pH and the lack of visible precipitation at the points of addition (the mixer walls are transparent). The four aqueous NaOH streams were injected into the seawater flow via four 1/4” Push-To-Connect NPTF threaded fittings tapped into the upstream end of the mixing tube. The pH of the modified seawater was constantly monitored by a SeaFET pH sensor configured with a flow through assembly fed by a small continuous flow teed off from the main flow after the mixer tube. A 10 m length of 2” hosing was used after the mixer to enable positioning of the outflow at a sufficient distance downstream (south) of the intake pump location to avoid re-uptake of modified seawater. The diameter of the hosing at the outflow was reduced from 2” to 1 1/2” to generate a slight back pressure and enable the small flow to the SeaFET sensor.

**Software and communications** A feedback system was implemented, whereby a Python program dynamically controlled the dosing rate in response to the continually monitored pH in the outflow. An automatic system shutdown in the case of low seawater flow (as measured by the flow meter) was also implemented as an additional safety feature. The dosing rate and sensor data were logged, with outputs visible in real time on the software user interface. Sampling and control data, including sample time, outlet seawater temperature, outlet seawater pH, seawater flow rate and NaOH dosing rate, were recorded every 3 seconds and logged locally. These data were time synchronised with the containerised sensor data using UTC, ensuring consistent timing across all logging systems.

### Ancillary observations - surface vehicle

A Maritime Robotics Otter Pro piloted surface vehicle with an AML Idronaut pH sensor (pH resolution 0.01, accuracy ± 0.1) was used to map the extent of the plume by driving it across the area of the outlet in a zig zag fashion of increasing distance to the outlet (see Fig. S5). The data from four tracks over the course of one hour on day 2 of the experiment was used to create a heat map of the plume, showing the change in pH(Autonomous Surface Vehicle), hereafter pH(ASV), over distance from the outlet. The data was linearly interpolated, and then a Gaussian filter was applied to reduce measurement noise while preserving the large-scale trends. This reduces fine-grained variations due to sensor noise or small-scale fluctuations, while still maintaining the integrity of broader gradients. We note that there is an offset between the pH(ASV) and the pH measured continuously in the container (see above). Because the pH(ASV) is used to illustrate the variation during the NaOH release, and as a proxy for the spatial dilution of the modified seawater plume, relative changes are sufficient, and a correction of these data was not undertaken.

**Table 1:** Mean parameter changes during each of the four experiments. Differences ($\Delta$) are based on pre-dosing background measurements taken immediately prior to dosing during each experimental day. The values of TCO$_2$ and Alk are from discrete samples, and the signal strength is computed on the basis of the NaOH addition and Alk samples at corresponding sample locations. NF1 = near field 1 position, NF2 = near field 2 position (see Fig. 5c) for sample locations. Parameter mean signals at each location as reported with standard deviation (stdev) in brackets, (-) indicates only one sample was taken and there is no stdev.

Experiment	Position	$\Delta$*p*CO$_2$	$\Delta$TCO$_2$	$\Delta$Alk	$\Delta$pH	Signal strength	Mean *p*CO$_2$	Mean TCO$_2$	Mean Alk	Mean pH
		$\mu$atm	$\mu$mol kg$^{-1}$	$\mu$mol kg$^{-1}$		$\%$	$\mu$atm	$\mu$mol kg$^{-1}$	$\mu$mol kg$^{-1}$	
**1**	Release	38	0	36	0.05	2.2	408 (25)	2044 (19)	2265 (19)	8.03 (0.03)
	NF1	28	-	-	0.03	-	407 (5)	-	-	8.01 (0.01)
	NF2	10	0	5	0.01	0.5	436 (3)	2058 (-)	2247 (-)	7.99 (0.00)
	Downstream	43	-2	29	0.05	2.8	403 (16)	2055 (-)	2271 (-)	8.03 (0.02)
**2**	Release	338	2	544	0.71	49.4	107 (57)	2059 (n=1)	2787 (-)	8.69 (0.03)
	NF1	15	-2	6	0.01	0.51	431 (8)	2055 (-)	2248 (-)	7.99 (0.00)
	NF2	10	4	9	0.01	0.8	435 (8)	2061 (-)	2251 (-)	7.99 (0.01)
	Downstream	54	6	49	0.06	4.5	391 (14)	2063 (-)	2291 (-)	8.05 (0.01)
**3**	Release	209	6	139	0.27	12.5	286 (32)	2077 (-)	2378 (-)	8.22 (0.02)
	NF1	-4	4	1	-0.01	0.1	498 (18)	2075 (-)	2240 (-)	7.65 (0.00)
	NF2	7	-3	0	0.01	0.0	488 (13)	2068 (-)	2239 (-)	7.69 (0.01)
	Downstream	8	0	7	0.01	0.6	487 (14)	2071 (-)	2246 (-)	7.97 (0.01)
**4**	Release	158	3	69	0.18	6.2	337 (26)	2074 (-)	2308 (-)	8.13 (0.04)
	NF1	-20	5	0	-0.02	0.0	515 (9)	2075 (-)	2239 (-)	7.94 (0.00)
	NF2	7	-9	0	0.01	0.0	486 (1)	2062 (-)	2239 (-)	7.96 (0.00)
	Downstream	62	-4	34	0.05	3.1	433 (17)	2067 (-)	2273 (-)	8.00 (0.02)
**Pre-experiment**							445 (20)	1898 (56)	2062 (61)	7.96 (0.01)
**Post-experiment**							458 (17)	1932 (23)	2109 (26)	7.97 (0.02)

## Supplementary Information

Supplementary Information.

## Data Availability

The datasets generated during and/or analysed during the current study are available from the corresponding author on reasonable request.
