# Supplementary material for: Alkalinity enhancement with sodium hydroxide in coastal ocean waters
Source: Sci Rep. 2025 Dec 12;16:1842. doi: 10.1038/s41598-025-31606-w (PMC12804671; doi:10.1038/s41598-025-31606-w)
Supplement: Supplementary file 1 — Supplementary Information. [file 41598_2025_31606_MOESM1_ESM.pdf]

Alkalinity enhancement with sodium hydroxide in coastal ocean waters

Cathryn A. Wynn-Edwards[1,*], Wayne Dillon[1], John Akl Uhri[1], Craig Neill[1], Harris Anderson[1], Hui Sheng Lim[2], Mathieu Mongin[1], and Elizabeth H. Shadwick[1]

[1]CSIRO, Environment, Hobart, 7000, Australia
[2]CSIRO, NCMI, Hobart, 7000, Australia
[*]Cathryn.Wynn-Edwards@csiro.au

Supplementary Information

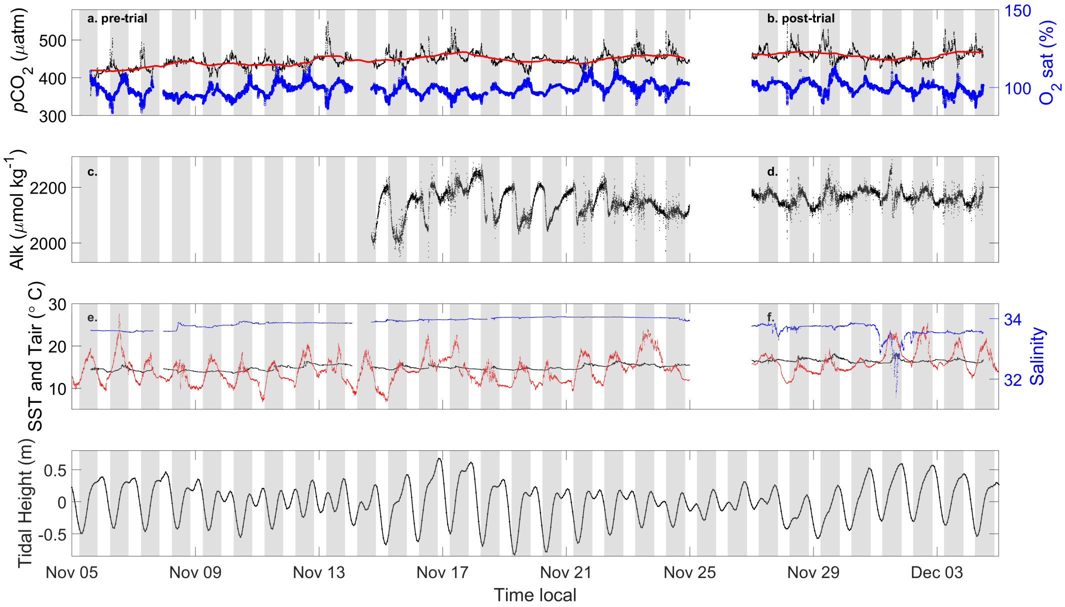

Figure S1. Baseline observations at the field trial site in Woodbridge, Tasmania. a., c. and e. pre-trial observations, b. d. and f. post-trial observations. a.-b. pCO_2 (black) with 24h running mean (red solid line) and dissolved oxygen saturation (O_2sat, in blue); c.-d. Alkalinity (Alk) calculated from sensor pCO_2 and pH. pH data was not measured prior to 14 Nov. e.-f. local sea surface temperature (SST, black) and air temperature (T_{air}, red) from a nearby weather station (Dennes Point, Fig. 1c.), and sea surface salinity (blue); g. tidal changes from a nearby gauge (Southport, Fig. 1b.). Grey shading in all panels represents daylight hours.

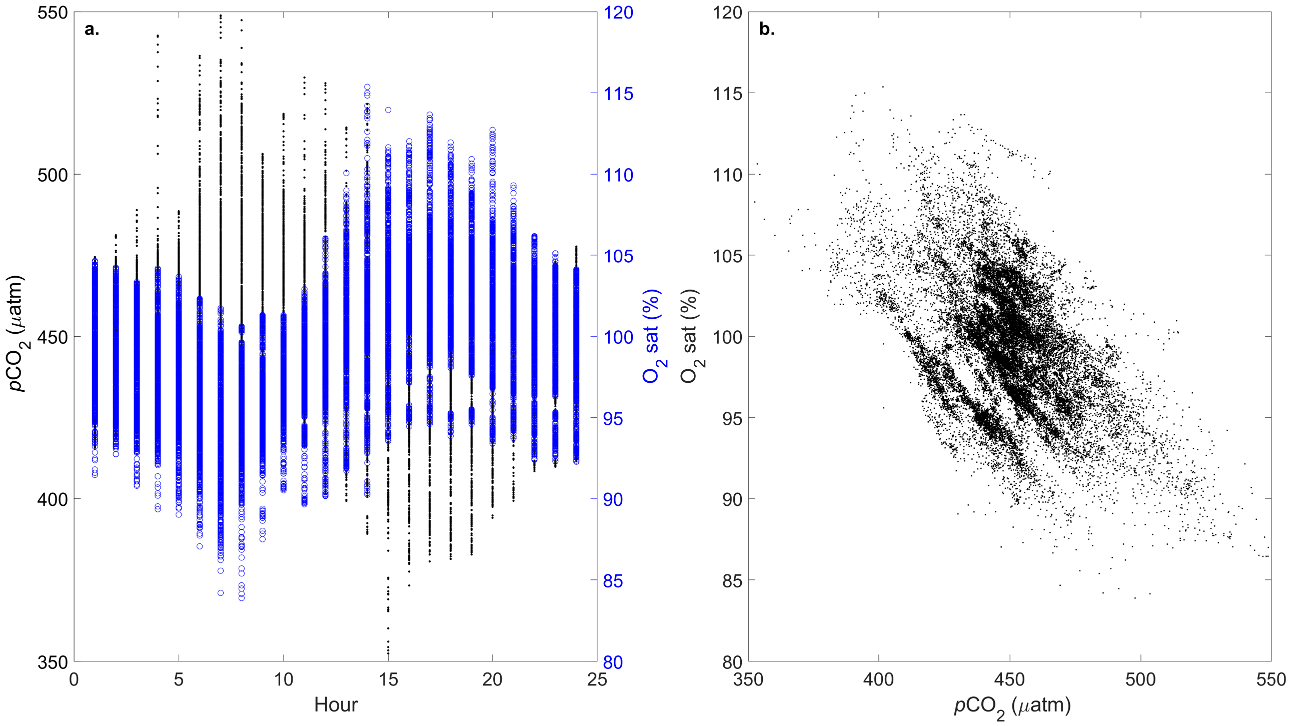

Figure S2. Variation in partial pressure of CO_2 (pCO_2) and dissolved oxygen saturation (O_2sat). a. diurnal variability of pCO_2 (black) and O_2 (blue) based on pre-trial observations collected over three weeks. b. the relationship between pCO_2 and O_2sat from observations collected as in panel a.

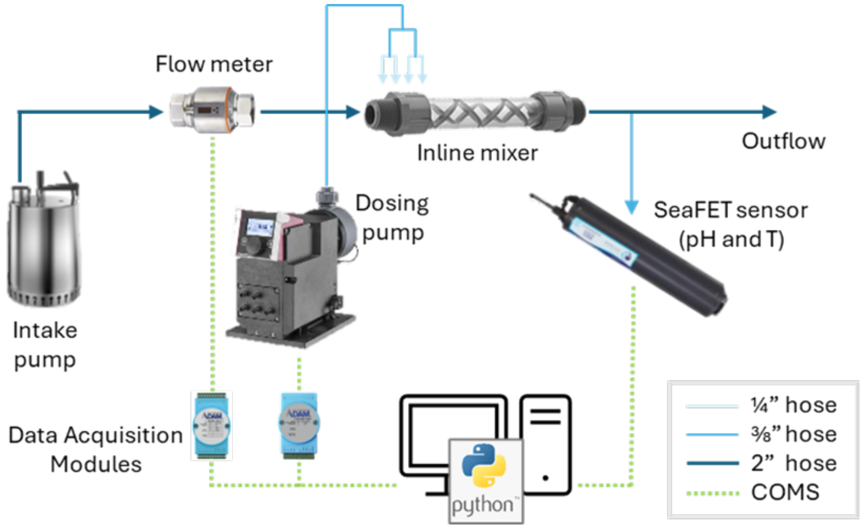

Figure S3. A schematic flow diagram of the inline NaOH dosing system. A list of components used to build the system is given in Table S3.

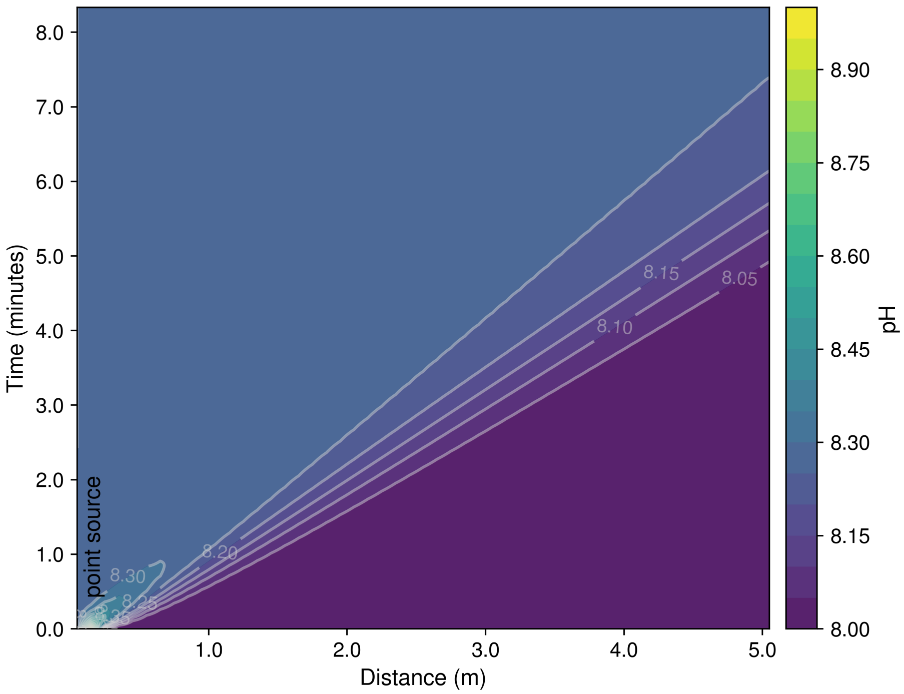

Figure S4. Idealised one dimensional advection-diffusion model for pH addition. The background pH is set to 8, signal is added at the point source (Time = 0, m = 0) at pH = 9. Diffusive flux is set to 1×10^{-6} m^2 s^{-1} and advection is set to 3 cm s^{-1}. Coloured surface represents the pH evolution of time and distance from the source, pale grey contours are the same as the surface.

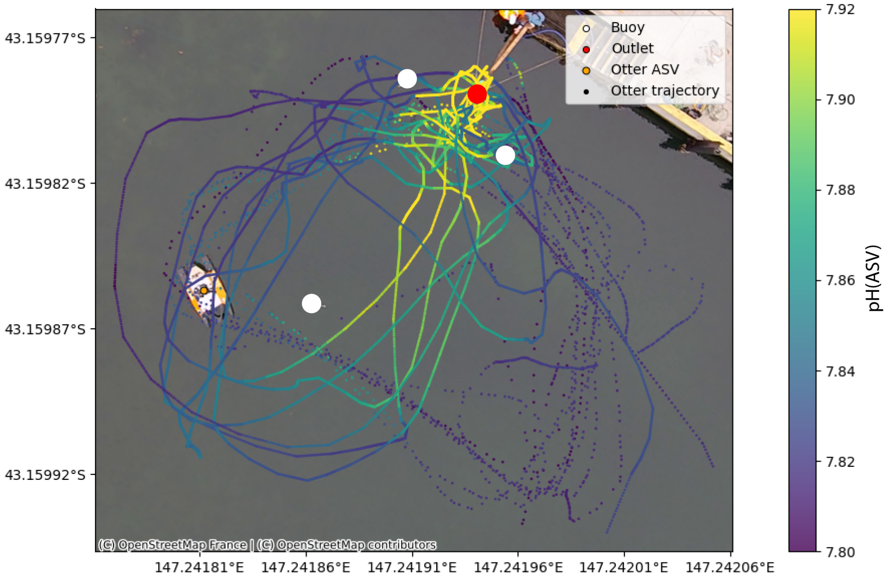

Figure S5. The plume of seawater modified with NaOH during Day 2 of the experiment. A piloted surface vehicle (Maritime Robotics Otter Pro) equipped with an AML Idronaut pH sensor was used to map the dispersion of the plume. The pH(ASV) observations were then interpolated to generate Figure 5c. in the main text (see Methods). The image in the background is a photograph of the field site taken with a drone; the jetty is visible in the top right corner. We note there is an offset between the pH(ASV) and the pH observations shown in Figs. 3 and 4 in the Main Text.

Table S1. Background measurements during the month of observations, not including the two experimental days: pCO_2 - partial pressure of CO_2 in μatm; pH; TCO_2 - Total inorganic carbon (calculated from sensor pH and pCO_2) in μmol kg^{-1}, Alk - alkalinity (calculated from sensor pH and pCO_2) in μmol kg^{-1}; SST - Sea surface temperature in °C; dissolved oxygen - O_2 in μmol L^{-1}; O_2sat - dissolved oxygen saturation in % and SAL - salinity. The minimum, maximum, and mean (using all observations), are given, as well as the night time (21:00 pm to 05:00 am) and day time (05:00 am to 21:00 pm) values, with associated standard deviations (stdev). Pre-trial values are shaded in grey, post-trial values are in white.

	**Overall**	**Overall**	**Night**	**Night**	**Day**	**Day**	**Exp hours (7am to 12pm)**	**Exp hours (7am to 12pm)**
	Min, max	Mean (stdev)	Min, max	Mean (stdev)	Min, max	Mean (stdev)	Min, max	Mean (stdev)
pCO_2	352, 549	445 (20)	414, 536	447 (14)	383, 549	445 (21)	401, 549	456 (22)
pCO_2	413, 543	458 (17)	420, 543	456 (11)	413, 528	462 (19)	442, 528	475 (17)
pH	7.91, 8.03	7.98 (0.01)	7.89, 8.00	7.98 (0.01)	7.91, 8.03	7.97 (0.02)	7.91, 7.99	7.96 (0.01)
pH	7.90, 8.04	7.98 (0.02)	7.90, 8.01	7.98 (0.01)	7.92, 8.04	7.97 (0.02)	7.92, 8.00	7.96 (0.02)
TCO_2	1781, 2127	1969 (50)	1824, 2107	1939 (69)	1781, 2217	1920 (60)	1824, 2127	1966 (52)
TCO_2	1869, 2135	1992 (23)	1869, 2087	1974 (19)	1903, 2135	1992 (22)	1915, 2091	1994 (22)
Alk	1927, 2294	2141 (54)	1971, 2288	2106 (77)	1927, 2294	2085 (67)	1978, 2294	2131 (57)
Alk	2016, 2354	2176 (26)	2016, 2263	2154 (22)	2074, 2354	2173 (26)	2074, 2268	2168 (26)
SST	13.47, 16.48	14.81 (0.60)	13.48, 16.05	14.75 (0.55)	13.47, 16.48	14.86 (0.64)	13.47, 15.72	14.47 (0.54)
SST	15.82, 18.35	16.52 (0.41)	15.83, 17.26	16.48 (0.27)	15.82, 18.35	16.54 (0.47)	15.91, 17.44	16.24 (0.23)
O_2	213.9, 285.4	248.0 (8.3)	217.0, 266.3	246.0 (5.9)	213.9, 285.4	249.0 (9.3)	213.9, 265.0	242.6 (7.3)
O_2	212.1, 268.4	244.5 (7.7)	217.3, 254.9	244.0 (5.4)	212.1, 268.4	243.1 (8.8)	212.1, 247.6	236.4, (6.0)
O_2sat	84, 115	99 (4)	87, 108	98 (3)	84, 115	99 (4)	84, 106	96 (3)
O_2sat	87, 114	101 (4)	89, 106	100 (3)	87, 114	100 (4)	87, 102	97 (2)
SAL	33.54, 34.07	33.90 (0.15)	33.55, 34.07	33.90 (0.15)	33.54, 34.07	33.92 (0.14)	33.54, 34.07	33.90 (0.16)
SAL	31.41, 33.89	33.62 (0.27)	32.82, 33.88	33.62 (0.19)	31.41, 33.89	33.58 (0.31)	32.45, 33.85	33.62 (0.17)

Table S2. Flow and dosing characteristics of the NaOH dosing system, including seawater (SW) flow rate, NaOH solution addition, and resulting Alk increase. Seawater density was assumed to be 1.025 kg L^{-1}.

**Experiment**	**Run time**	**NaOH Dosing Rate**	**Total NaOH Dosing Volume**	**Moles of 1 M NaOH**	**Mean SW Flow Rate (stdev)**	**Total SW Volume**	**Total Alk increase**
	min	L min^{-1}	L	mol	L min^{-1}	L	μmol kg^{-1}
1	92	(0.2-0.3)	21.7	21.7	221 (4)	20426	1036
2	76.8	0.3	19.2	19.2	222 (1)	17000	1101
3	122.2	0.3	30.5	30.5	219 (3)	26792	1112
4	103.1	0.3	25.8	25.8	219 (6)	22541	1115

Table S3. Components used in the NaOH dosing system (see Fig. S3).

**Component**	**Supplier and model**	**Details**
Submersible pump	Grundfos, UNILIFT AP12.50.11.1	220-240V, 8.5A. 2” female BSP thread
Flow meter	IFM, SM2604	Inline. 2” female NPT threads. COMS: analogue output
Dosing pump	Grundfos, SMART Digital XL – DDA	0 – 200 L/hr dosing rate. COMS: analogue input
Inline mixer	McMaster-Carr, PN: 35385K27	Inline. 2” male NPT threads
SeaFET	Satlantic	COMS: serial USB
Push-To-Connect ’Y’ fitting	McMaster-Carr, PN: 9087K58	3/8” hosing
Push-To-Connect fittings	McMaster-Carr, PN: 9087K11	Male NPT thread, 1/4” hosing
Tubing (3/8” OD)	McMaster-Carr, PN: 2129T17	Hard, chemical resistant
Tubing (1/4” OD)	McMaster-Carr, PN: 2129T16	Hard, chemical resistant
ADAM-4017	Advantech	Data acquisition (flow meter)
ADAM-4021	Advantech	Data acquisition (dosing pump)
